# Agranulocytosis Associated With Spironolactone Therapy: A Case Report

**DOI:** 10.4021/wjon356w

**Published:** 2011-10-28

**Authors:** Adam A. Fershko, Jennifer A. Neely, Alejandro R. Calvo

**Affiliations:** a Kettering Medical Center, Internal Medicine Residency Program, Department of Medical Education, Dayton, Ohio, USA; b Dayton Cancer Center – Kettering Health Network, Dayton, Ohio, USA; c Kettering Collegeof Medical Arts, 3737 Southern Blvd, Kettering, Ohio 45429, USA

**Keywords:** Drug induced agranulocytosis, Agranulocytosis

## Abstract

Herein, we report a case where agranulocytosis occurred after spironolactone administration. Patient presented with non-descript constitutional symptoms suggestive of a viral etiology associated to new onset agranulocytosis with neutrophilic maturation arrest on bone marrow biopsy. Patient’s medical history included chronic liver disease as well as new onset acute renal insufficiency. Upon review of patient’s medications, initiation of spironolactone was noted 4 weeks prior to admission. Few cases of agranulocytosis secondary to spironolactone have been reported in the literature, most of which were also in association with both renal insufficiency and chronic liver disease. Discontinuation of spironolactone resulted in normalization of granulocyte count within 3 weeks. As patients with chronic liver disease are frequently given spironolactone, we recommend monitoring blood counts 4 - 8 weeks following initiation of therapy to detect and treat this potentially life threatening complication.

## Introduction

Drug-induced agranulocytosis is a challenging diagnosis; it requires a high level of clinical suspicion and a detailed history of medication intake prior to the episode. We report a rare complication of spironolactone causing reversible agranulocytosis in a patient with cryptogenic liver cirrhosis. This adverse effect is seen more commonly in patients with some degree of renal and/or liver dysfunction as well as advanced age. As this drug is commonly used in patients with cirrhosis with ascites, we recommend monitoring a complete blood count periodically, at least for the first 2 months of therapy.

## Case Report

**Figure 1:**
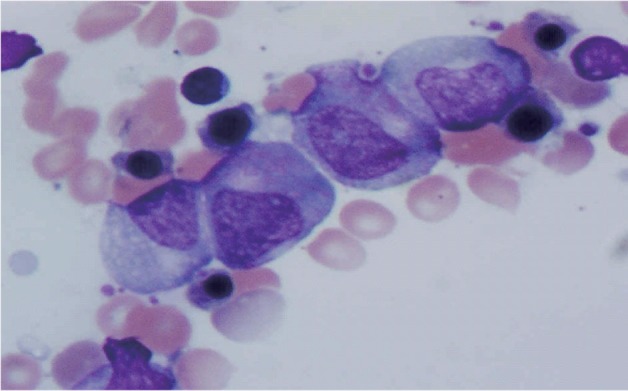
Patient’s bone marrow demonstrating promyelocyte-to-myelocyte maturation arrest.

**Figure 2:**
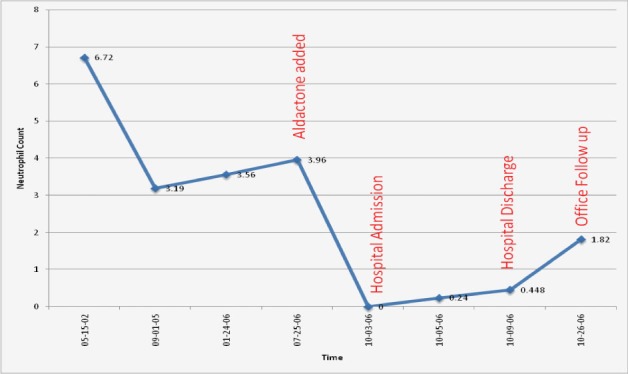
ANC before, during and after spironolactone exposure.

A 75-year-old Caucasian female with known ischemic heart disease and cryptogenic cirrhosis presented to the hospital with complaints of chest pain, shortness of breath, malaise and increased weakness. Cardiac workup was negative. Patient was found on presentation to have severe leukopenia with agranulocytosis and relative lymphocytosis. Records revealed that her white count had been normal only three months earlier. Patient’s presenting white count was 3,000/mm^3^ with 64% lymphocytes, 29% monocytes but no segmented neutrophils present on differential count. A bone marrow biopsy showed neutrophilic maturation arrest at the promyelocyte to myelocyte stage (Fig. 1). Levels of B12 and folate were normal. Spironolactone was started approximately 1 month prior to her admission for treatment of ascites related to cryptogenic cirrhosis. Blood counts done after discontinuation of spironolactone showed evidence of complete neutrophil count recovery within 21 days (Fig. 2).

## Discussion

Case reports have demonstrated that spironolactone can cause agranulocytosis [1, 2, 3]. Advanced age, hepatic and renal impairment and concurrent medications have all been identified as contributing to the likelihood of this adverse effect.

Agranulocytosis is relatively rare with only 1 - 5 cases per million per year [4]. Medications can be the etiology in as many as 70% of cases [5]. Common drugs implicated include clozapine, antithyroid drugs, sulfasalazine, and ticlopidine. In a study done in the Netherlands where patients were admitted for agranulocytosis, discovered that three drugs – methimazole, sulfasalazine and trimethoprim-sulfamethoxazole – were found to be the cause in 42% of all cases [6]. There are two basic mechanisms by which drugs are proposed to cause agranulocytosis. One postulated mechanism is that of immune mediated destruction of circulating neutrophils by drug induced antibodies. The other proposed mechanism is that there can be direct toxin effect on the bone marrow precursor cells. It is the latter mechanism that is likely the etiology for spironolactone-induced agranulocytosis as evidenced by the arrested development of our patient’s myelocytes on bone marrow evaluation. Six cases of spironolactone induced agranulocytosis dating from 1984 thru to 2003 have been reported [7], of which all demonstrated resolution of agranulocytosis following cessation of the offending agent. It does appear that agranulocytosis can be a severe side effect secondary to spironolactone and, although rare, does appear to be relatively more likely in patients that have both renal insufficiency and liver disease. Thus, it may behoove the physician starting this medication to monitor blood counts in patients recently started on this medication especially if they have the aforementioned comorbidities.

## References

[1] Whitling AM, Pergola PE, Sang JL, Talbert RL (1997). Spironolactone-induced agranulocytosis. Ann Pharmacother.

[2] Hsiao SH, Lin YJ, Hsu MY, Wu TJ (2003). Spironolactone-induced agranulocytosis: a case report. Kaohsiung J Med Sci.

[3] Kelly JP, Kaufman DW, Shapiro S (1991). Risks of agranulocytosis and aplastic anemia in relation to the use of cardiovascular drugs: The International Agranulocytosis and Aplastic Anemia Study. Clin Pharmacol Ther.

[4] Kaufman DW, Kelly JP, Issaragrisil S, Laporte JR, Anderson T, Levy M, Shapiro S (2006). Relative incidence of agranulocytosis and aplastic anemia. Am J Hematol.

[5] Kaufman DW, Kelly JP, Jurgelon JM, Anderson T, Issaragrisil S, Wiholm BE, Young NS (1996). Drugs in the aetiology of agranulocytosis and aplastic anaemia. Eur J Haematol Suppl.

[6] van der Klauw MM, Goudsmit R, Halie MR, van't Veer MB, Herings RM, Wilson JH, Stricker BH (1999). A population-based case-cohort study of drug-associated agranulocytosis. Arch Intern Med.

[7] Andersohn F, Konzen C, Garbe E (2007). Systematic review: agranulocytosis induced by nonchemotherapy drugs. Ann Intern Med.

